# Long noncoding RNA uc007nnj.1 mediates neuronal death induced by retinal ischemia/reperfusion in mice via the miR-155-5p/Tle4 axis

**DOI:** 10.1186/s10020-022-00591-5

**Published:** 2023-01-18

**Authors:** Yuqing Feng, Jinfang Lu, Xujun Peng, Yanni Ge, Ran Zhang, Huiling Li

**Affiliations:** 1 Department of Ophthalmology, The Second Xiangya Hospital, Central South University, Changsha, 410011 Hunan China; 2 Hunan Clinical Research Center of Ophthalmic Disease, Changsha, 410011 Hunan China

**Keywords:** lncRNA uc007nnj.1, I/R, RGCs, Apoptosis, miR-155-5p

## Abstract

**Background:**

Retinal ganglion cells (RGCs) apoptosis is a vital manifestation of retinal ischemia/reperfusion (I/R) injury, yet the underlying mechanisms are not well understood. The contribution of long noncoding RNAs (lncRNAs) to this cellular process is currently being explored. Based on a lncRNA chip assay, we aimed to investigate the role of lncRNA uc007nnj.1 in the pathological process of ischemia-induced RGCs apoptosis.

**Methods:**

Hank’s balanced salt solution containing 10 µM antimycin A and 2 µM calcium ionophore for 2 h to construct an ischemic model in RGCs, and elevation of intraocular pressure to 120 mm Hg for 1 h was used to construct a mouse model of retinal I/R injury.

**Results:**

In this study, lncRNA uc007nnj.1 was highly upregulated in response to I/R injury in RGCs and mouse retinas. In addition, lncRNA uc007nnj.1 knockdown reduced retinal neuronal cell apoptosis in vitro and in vivo and significantly improved retinal function.

**Discussion:**

Mechanistically, the results demonstrated that lncRNA uc007nnj.1 acts as ceRNA competitively binding miR-155-5p, thereby enhancing the expression levels of Tle4, thus aggravating ischemia-related apoptosis in RGCs.

**Conclusions:**

Finally, our study identifies the lncRNA uc007nnj.1/miR-155-5p/Tle4 axis as a potential target for the prevention of I/R-induced retinal neuronal death.

**Supplementary Information:**

The online version contains supplementary material available at 10.1186/s10020-022-00591-5.

## Background

Retinal ischemia/reperfusion (I/R) is a common pathophysiological element of various vision-disabling ocular diseases, such as glaucoma, diabetic retinopathy, and ischemic optic neuropathy (Kergoat et al. 2006; Osborne et al. 2004). Retinal ganglion cells (RGCs) apoptosis is pivotal in ischemia–reperfusion injury (Zhou et al. 2019). Over recent decades, the molecular mechanisms of apoptosis in RGCs have been investigated in several studies, including the endoplasmic reticulum, oxidative stress, and mitochondrial dysfunction (Almasieh et al. 2012; Li et al. 2014). However, the pathology that leads to ganglion cell death is still under considerable study. A better understanding of the underlying pathomechanisms involved in RGCs apoptosis is crucial for developing novel diagnostic and therapeutic strategies to alleviate retinal ischemic injury and prevent vision loss.

Long noncoding RNAs (lncRNAs) are noncoding molecules longer than 200 nucleotides with limited phylogenetic conservation and are characterized by tissue/organ-specific expression profiles (Yao et al. 2019). An increasing number of lncRNAs have recently been identified to be associated with many pathological processes, including inflammation and cell apoptosis (Radhakrishnan and Kowluru 2021; Zhang et al. 2021; Qian et al. 2019). It is known that lncRNAs contribute to many ocular disorders such as glaucoma, corneal diseases, cataracts, and diabetic retinopathy (Zhang et al. 2019; Shen et al. 2020; Sun et al. 2019; Tu et al. 2020; He et al. 2021). To investigate the potential role of lncRNAs in RGCs apoptosis, we previously assessed the lncRNA profile of RGCs using ChIP assays and identified 416 lncRNAs (fold change greater than four) elevated in RGCs in the ischemia group versus the control group (Ge et al. 2020). Among them, we have reported the function of lncRNA Ttc3-209, which promotes apoptosis in RGCs following ischemic injury (Zhang et al. 2021). However, for many other lncRNAs, the biological functions and regulatory mechanisms remain largely unclarified. Here, we aimed to explore more expression patterns and new molecular mechanisms of these lncRNAs with high fold changes, which can contribute to a better understanding of the pathophysiological mechanisms underlying RGCs apoptosis.

In the present study, we focused on a candidate lncRNA with high expression (fold change of 8.59) (Additional file 1: Table S1) under ischemic conditions, lncRNA uc007nnj.1, derived from the Rho GTPase activating protein 5 (Arhgap5, Gene ID: 11855) gene, for further cellular and animal studies. We found that knockdown of lncRNA uc007nnj.1 ameliorated ischemia-induced apoptosis in RGCs. Moreover, lncRNA uc007nnj.1 acts as a ceRNA to regulate the expression of Tle4 by decoying miR-155-5p, thus mediating retinal neuronal death. The mechanism through which lncRNA uc007nnj.1 mediates retinal I/R injury suggests that inactivation of this candidate lncRNA could be an interesting strategy for retinal neuroprotection.

## Methods

### Animal care and use

Ten- to twelve-week-old male wild-type C57BL/6J mice were obtained from the Experimental Animal Center of Central South University. Mice were maintained in a specific pathogen-free (SPF) facility under a 12-h light/dark cycle with free access to food and water. All animal procedures were approved by the Institution of Animal Care and Use Committee of the Second Xiangya Hospital and adhered to the Guidelines for the Care and Use of Laboratory Animals. The retinal I/R model was established as previously reported (Li et al. 2014; Ge et al. 2020; Kim et al. 2013). Briefly, mice were randomly assigned to groups (n = 6 per group) and then sedated by intraperitoneal injection of sodium pentobarbital. Next, a 33-gauge infusion needle containing 0.9% NaCl was inserted into the anterior chamber to maintain the intraocular pressure at 120 mmHg (measured with a TonoLab tonometer). Transient retinal ischemia was achieved for 60 min. Then, the needle was removed, and reperfusion was initiated. The contralateral eye was cannulated to maintain a normal IOP and served as a nonischemic control (Li et al. 2014; Deng et al. 2020; Souza Monteiro de Araujo et al. 2020). lncRNA uc007nnj.1 siRNA or scramble or lncRNA uc007nnj.1 plasmid or vector was delivered directly into the vitreous chamber of mice (1 μL per injection, 1 μg/μL) 24 h before the retinal I/R model was constructed as previously described (Zhang et al. 2021; Kleinman et al. 2008; You et al. 2017; Hou et al. 2016).

### Isolation and culture of mouse primary RGCs

Primary retinal ganglion cells were isolated from the retinas of 1- to 4-days-old neonatal mice according to the previously published protocols (Zhang et al. 2021; Ge et al. 2021; Huang et al. 2003; Chintalapudi et al. 2016). Routine asepsis was performed. Briefly, 24-well plates were precoated with poly-d-lysine (P6407; Sigma, St. Louis, MO, USA) and laminin (L-6274; Sigma) 1 day in advance. Then, 75 cm^2^ cell culture dishes were coated with purified donkey anti-rabbit IgG (H&L) (ab150075; Abcam, Cambridge, MA) for anti-macrophage elution, and 100 cm^2^ plates were coated with donkey anti-rat IgG (H&L) for immobilization of Thy1.2 antibody (BE0066; Bio X Cell, West Lebanon, NH, USA). All of the above plates were incubated overnight at 4 °C. After dissection of the retina and a wash with DPBS at 37 °C for 15 min in papain (G8430; Solarbio, Beijing, China) containing DNase I (Sigma, St. Louis, MO), the papain solution was carefully aspirated, and Lo Ovo solution containing anti-macrophage antibody (AIA31240; Accurate Chemical, Westbury, NY, USA) was added. The cells were incubated for 5 min at RT. The supernatant was centrifuged and discarded, the cells were resuspended with panning buffer, and the cell suspension was transferred into a preprepared purified donkey anti-rabbit IgG (H&L)-coated culture dish and incubated at room temperature for 40 min. Nitex mesh (352,350; BD Biosciences, Franklin Lakes, NJ) was used to filter the cell suspension, which was transferred into a culture dish precoated with an anti-Thy1.2 antibody (M7898; Sigma) and incubated for 1 h. Nonadherent cells were removed, and the RGCs in the culture dish were digested with 4 mL of trypsin/Earle's balanced salt solution (EBSS) for 5 min. After centrifugation, the cells were resuspended in a complete medium and grown in 24-well plates. RGCs were cultured in a humidified atmosphere at 37 °C with 5% CO_2_ and 95% O_2_; 50% of the medium was replaced with a prewarmed complete growth medium every 3 days.

### In vitro ischemia simulation

We established an in vitro mimic of ischemic injury in RGCs according to our previously applied calcium ionophore/ATP depletion injury model (Zhang et al. 2021; Ge et al. 2020; Lee and Emala 2002). When RGCs were confluent over 90% of the plate, ischemia was simulated for 2 h by changing the medium to Hanks’ balanced salt solution (HBSS) with 10 mM antimycin A (a complex III inhibitor of mitochondrial electron transport; ab141904; Abcam, Cambridge, MA, US) and 2 mM calcium ionophore (A2318; Aladdin, Shanghai, China), which were dissolved in dimethylsulfoxide (DMSO). The complete growth medium was reapplied, and the cells were sustained for 0–4 h. For gene knockdown and overexpression experiments, 24 h before ischemic injury, when the density of the RGCs on the 24-well plate reached 80% to 90%, the cells were transfected lncRNA uc007nnj.1 siRNA/plasmid, miR-155-5p mimic or inhibitor, Tle4 siRNA, and scramble siRNA (Ribobio) using Lipofectamine 2000. After 6–8 h, the transfection solution was removed and replaced with a complete growth medium. Immunofluorescence staining of Tuj1 (1:500, GB11139; Servicebio, Wuhan, China) was used to identify the purity of RGCs.

### Fluorescence In Situ Hybridization (FISH)

A Fluorescence In Situ Hybridization Kit and probes were purchased from RiboBio Corporation (C10910; Guangzhou, China). In short, paraformaldehyde-fixed RGCs were prepared and then hybridized with a Cy3-labeled lncRNA uc007nnj.1 probe and FAM-labeled miR-155-5p probe. The nuclei were counterstained with 4′,6-diamidino-2-phenylindole (DAPI), and fluorescence images were taken via laser-scanning confocal microscopy (TCS SP5; Leica, Wetzlar, Germany). 18 s and U6 served as positive cytoplasmic and positive cytosolic controls, respectively.

### Luciferase reporter assay

The wild-type or mutant sequence of the Tle4 and lncRNA uc007nnj.1 3′-UTR (3′-untranslated region) was inserted into a pmirGLO vector and named Tle4 3′UTR WT, Tle4 3′UTR MUT, uc007nnj.1 WT or uc007nnj.1 MUT, as appropriate. RGCs were cotransfected with miR-155-5p mimic or miR-155-5p inhibitor or scramble and these reporter plasmids. After 48 h, the cells were collected with Passive Lysis Buffer, and a dual luciferase assay system was used to detect firefly and Renilla luciferase activities (Promega) according to the manufacturer’s instructions. Firefly luciferase activities were normalized according to Renilla luciferase levels. All plasmids were constructed by RuQi Biotechnology (Guangzhou, Guangdong Province, China).

### Western blotting

According to our previous studies, total protein was extracted from retinal tissues or RGCs using lysis buffer (Zhang et al. 2017a, b). Protein concentrations were measured using a NanoPhotometer N50 Touch spectrophotometer (IMPLEN, Heidelberg, Germany). Equal amounts of protein per lane (30 µg) were run on a 10% SDS–PAGE gel and then transferred to polyvinylidene difluoride membranes. The blots were probed with primary antibodies against caspase-3 ([1:1000], 9662; CST, Danvers, MA, US), cleaved caspase-3 ([1:1000], 9661; CST, Danvers, MA, US), Tle4 (15140, [1:1500]; Novusbio, Minneapolis, MN, US), and β-actin (66009-I-Ig, [1:1000]; Proteintech, Rosemont, IL, US) followed by incubation with secondary antibodies (goat anti-rabbit IgG (H + L) HRP [1:5000] or goat anti-mouse IgG (H + L) HRP [1:5000], Affinity, Cincinnati, OH, US). The expression of target proteins was normalized to the corresponding β-actin expression level in the same sample and quantified using ImageJ software (National Institutes of Health, Bethesda, MD, USA). Each immunoblot was repeated three times to confirm the results.

### Reverse transcription-quantitative real-time PCR (RT-qPCR)

According to the manufacturer’s protocol, total RNA was isolated from retinas or cells using TRIzol reagent (Invitrogen, Carlsbad, CA, USA). Complementary DNA (cDNA) synthesis was performed using a Prime Script RT Reagent Kit and gDNA Eraser Kit (RR047A; TaKaRa, Tokyo, Japan). RT-qPCR was performed using SYBR Green (K0221; Thermo Fisher Scientific, Waltham, MA, USA), and the results were quantitated with StepOne Software (Applied Biosystems, Carlsbad, CA, USA). β-Actin was used as a standard for each sample to determine relative expression levels. The primer sequences for lncRNA uc007nnj.1, miR-155-5p, and Tle4 are shown in Additional file 1: Table S2. Relative RNA levels were calculated using the 2^−ΔΔCt^ method.

### Flow cytometry (FCM)

RGCs were trypsinized, washed twice with cold PBS, and then resuspended in 1× Binding Buffer. According to the manufacturer’s protocol, 100 µL of each solution was transferred to a 1.5 mL Eppendorf tube and then incubated with YF 488-annexin V and PI (Everbright, Suzhou, China, USA) in the dark for 15 min at RT. Finally, 400 μL of 1× binding buffer was added to the tube. Flow cytometry was then performed within 1 h using a BD FACSCalibur flow cytometer (San Diego, CA, USA). The results were obtained using FlowJo V10 software (BD, San Diego, CA, USA).

### Immunofluorescence and TUNEL assays

RGCs were identified and quantified utilizing Tuj1 immunofluorescence staining. Briefly, mice were killed 24 h after reperfusion. Eyes were enucleated within 10 min of death and fixed in 4% paraformaldehyde for 1 h. After carefully removing the anterior section, we placed the eyecup in FAS eye-fixation buffer (Servicebio; G1109) at 4 °C for 24 h and dehydrated it in a 30% source solution. Following fixation, some retinas were evaluated as flat mounts; the others were embedded in optimum cutting temperature compound (OCT) to obtain 10-µm-thick cryosections using a freezing microtome. For immunofluorescence analysis in retinal flat-mount and frozen sections, the retina was permeabilized and blocked with 10% Triton X-100 and 10% BSA for 1 h and immunolabeled with primary antibody (Tuj1 [1:500], GB11139; Servicebio, Wuhan, Hubei Province, China) overnight at 4 °C, followed by a 2-h incubation with a secondary antibody (goat anti-rabbit IgG (H + L) Alexa Fluor 488 [1:1000], ab150077; Abcam) in the dark at room temperature (RT). After being washed with PBS, the retina was stored in the dark at 4 °C until microscopic observation. For TUNEL staining, a terminal-deoxy-transferase-mediated 20-deoxyuridine, 50-triphosphate (dUTP) nick end-labeling (TUNEL) (#1684795; Roche, Basel, Switzerland) assay was performed in cryosections according to the manufacturer’s instructions. The sections were incubated with TUNEL reaction solution (including 50 µL Enzyme Solution [TdT] and 450 µL Label Solution [fluorescein-dUTP]) at 37 °C for 1 h and then mounted. DAPI staining was performed to visualize nuclei. Images were taken with a fluorescence microscope (Leica DMI3000B) in two different regions at distances of 1 and 2 mm from the disc of each quadrant in the retina to analyze the mean number of surviving RGCs. Four randomly selected fields were analyzed in each section.

### Electroretinography (ERG)

The b-wave amplitudes in ERG have been considered a sensitive parameter for detecting the degree of retinal injury induced by ischemia (Dang and Zhang 2018; Grozdanic et al. 2003; Ji et al. 2021). Therefore, scotopic ERG was recorded in mice after 6 h of dark exposure as previously described. The animals were anesthetized by intraperitoneal injection of sodium pentobarbital. After topically applying 0.4% levofloxacin and 1% tropicamide ophthalmic solution, we placed five electrodes on the subcutaneous tissue by the tails, beneath each eye, and at the apex of the corneas in both eyes. Flash ERG was recorded at an intensity of 3.0 cd·s/m^2^ using a Ganzfeld Q450 (ROLAND ELECTRONIC, Keltern, Germany) system. The evaluated b-waves were analyzed with RETI-Port/Scan 21 software (ROLAND ELECTRONIC, Keltern, Germany).

### Statistical analysis

All the results are presented as the mean ± SD of six independent experiments. Student’s *t* test was used to compare the means between two groups. One-way ANOVA followed by Tukey’s post-hoc test was used to compare multiple treatment groups. The data were considered to be statistically significant when the p value was less than 0.05. All statistical analyses were performed using SPSS software and GraphPad Prism software (GraphPad Software, La Jolla, CA, USA).

## Results

### Ischemia–reperfusion injury induced lncRNA uc007nnj.1 expression in vivo and in vitro

**Fig. 1:**
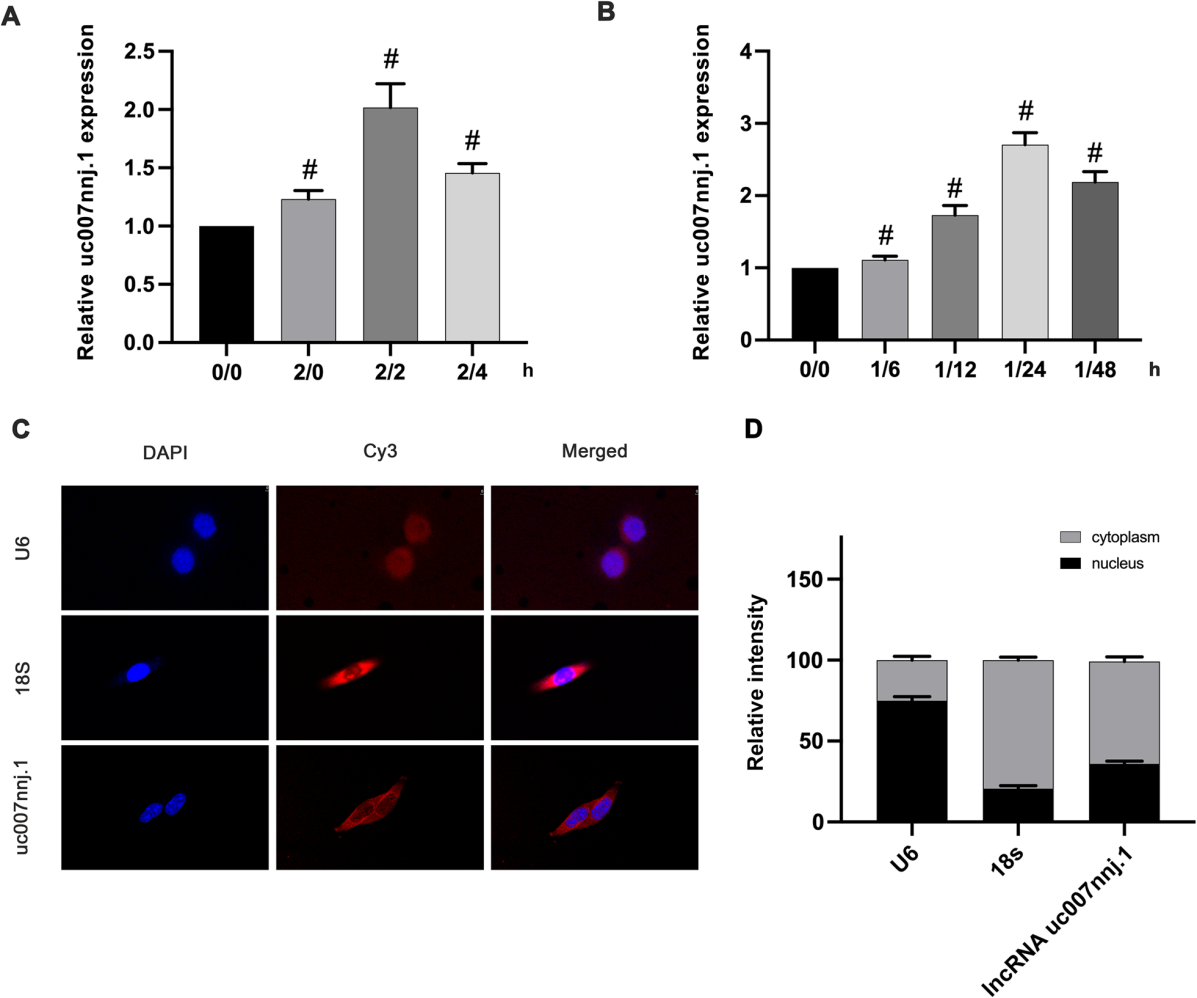
Ischemia–reperfusion injury induced lncRNA uc007nnj.1 expression of in vivo and in vitro. Cultured primary mouse RGCs were treated with or without Ca^2+^ (2µM) and antimycin (10 µM) in HBSS (ATP and glucose depletion) at indicated time periods (ischemia/reperfusion for 0/0 h, 2/0 h, 2/2 h, and 2/4 h, respectively). Reverse transcription-quantitative real-time PCR (RT-qPCR) analysis was used to detect the expression of lncRNA uc007nnj.1. **B** The intraocular pressure was elevated to 120 mmHg for 1 h to induce transient retinal ischemia and then exposed to reperfusion for 6, 12, 24, and 48 h in C57BL/6J mice. The expression levels of lncRNA uc007nnj.1 in the retinas were detected by RT-qPCR. **C** Representative FISH images of the location of lncRNA uc007nnj.1 (red) in RGCs. U6 and 18 s were used as nuclear and cytoplasmic markers, respectively. Scale bar: 10 µm. **D** Relative intensity analysis of FISH images. Data are expressed as mean ± SD of six independent experiments; ^#^p < 0.05 versus 0/0 h or sham group

By using a ChIP assay, we previously found that the candidate lncRNA, uc007nnj.1 was differentially expressed in RGCs under ischemic injury (Ge et al. 2020).To obtain a better understanding of the role of lncRNA uc007nnj.1, we initially used in vivo and in vitro ischemic experiments to investigate its expression profile. We first mimicked the ischemic model in primary RGCs by applying calcium ionophore/ATP depletion injury, followed by reperfusion. RT-qPCR data revealed upregulated expression levels of lncRNA uc007nnj.1 at 0 h after reperfusion, reaching a peak at 2 h, and then gradually declining at 4 h after reperfusion (Fig. 1A). Meanwhile, we found that the expression of retinal lncRNA uc007nnj.1 was increased 6 h after reperfusion and peaked at 24 h after reperfusion in mouse retinas with I/R injury (Fig. 1B). RNA fluorescence in situ hybridization (FISH) assays further indicated that lncRNAuc007nnj.1 was clearly expressed in the cytoplasm of RGCs (Fig. 1C, D). These data suggest that lncRNA uc007nnj.1 may be an ischemia-related factor.

### lncRNA uc007nnj.1 deletion alleviated ischemia-induced neuronal apoptosis

**Fig. 2:**
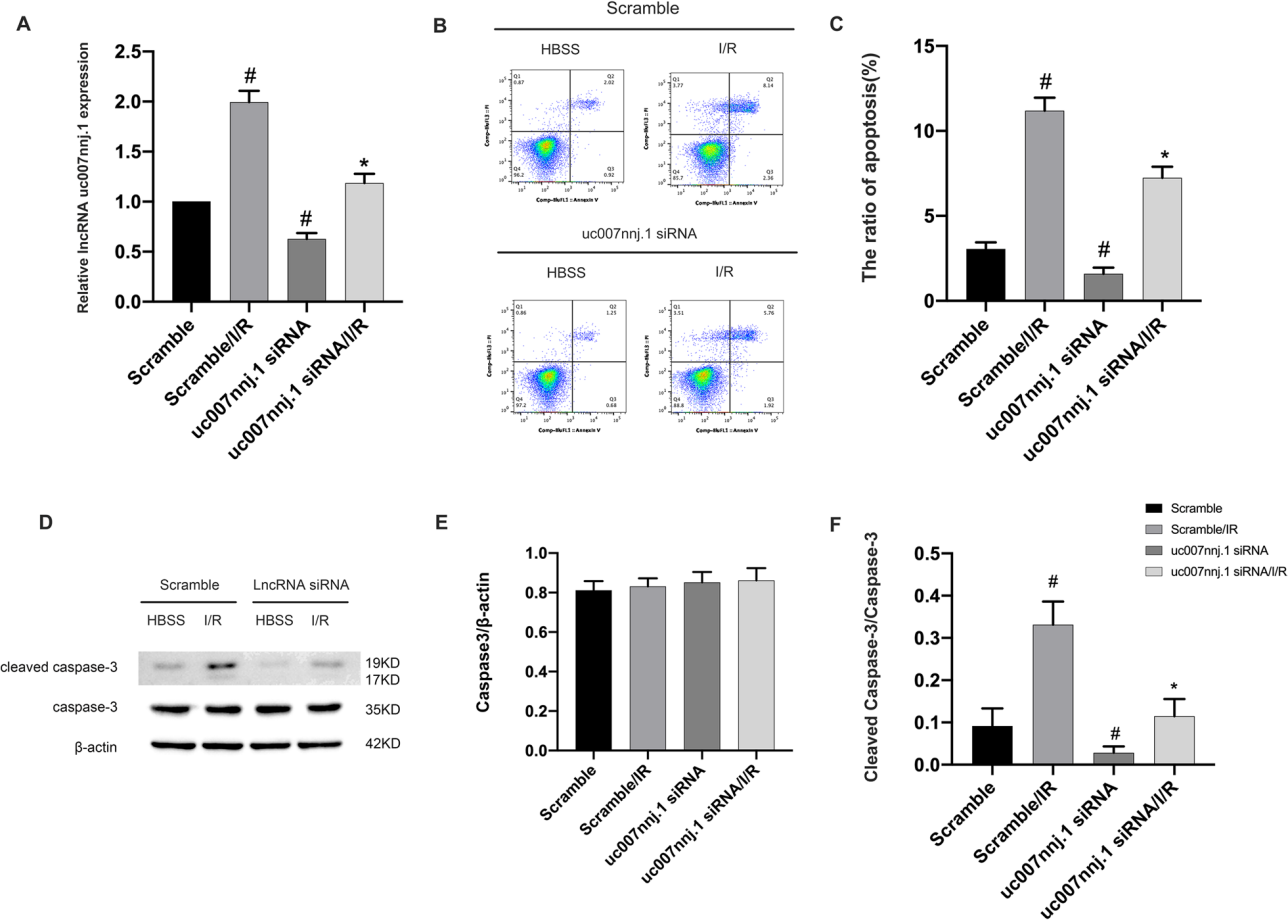
lncRNA uc007nnj.1 deletion alleviated ischemia-induced neuronal apoptosis. RGCs were transfected with 50 nM scramble siRNA or lncRNA uc007nnj.1 siRNA for 24 h and then treated with or without I/R injury. **A** RT-qPCR analysis of the expression of lncRNA uc007nnj.1. **B**, **C** Measurement of total apoptosis by flow cytometry in RGCs. The Q1 area represents necrotic cells, the Q2 area represents late apoptotic cells, the Q3 area represents early apoptotic cells, and the Q4 area represents normal cells. **D** Representative image showing a Western blot of caspase-3 and cleaved caspases-3. **E**, **F** Quantitative analysis of immunoreactive bands of caspase-3 (**E**) and cleaved caspase-3 (**F**). Data are expressed as mean ± SD (n = 6). ^#^*p* < 0.05, scramble with I/R group or lncRNA uc007nnj.1 siRNA group versus scramble group; *p < 0.05, lncRNA uc007nnj.1 siRNA with I/R group versus scramble with I/R group

TO detect the role of lncRNA uc007nnj.1 in retinal neurodegeneration, we deleted the lncRNA by transfecting RGCs with specific small interfering RNA (siRNA) before they were subjected to mimicked ischemic treatment. RT-qPCR analysis indicated that the specific siRNA obviously suppressed lncRNA uc007nnj.1 levels under both basic and ischemic conditions (Fig. 2A). Furthermore, transient gene silencing of lncRNA uc007nnj.1 notably alleviated apoptosis (Fig. 2B, C) and reduced the accumulation of active caspase-3 in RGCs under ischemic injury (Fig. 2D–F). These results suggest that lncRNA uc007nnj.1 reduction alleviated ischemia-induced neuronal apoptosis, which further supports the notion that it has a proapoptotic role in RGCs.

### Overexpression of lncRNA uc007nnj.1 aggravated ischemia-induced neuronal apoptosis

**Fig. 3:**
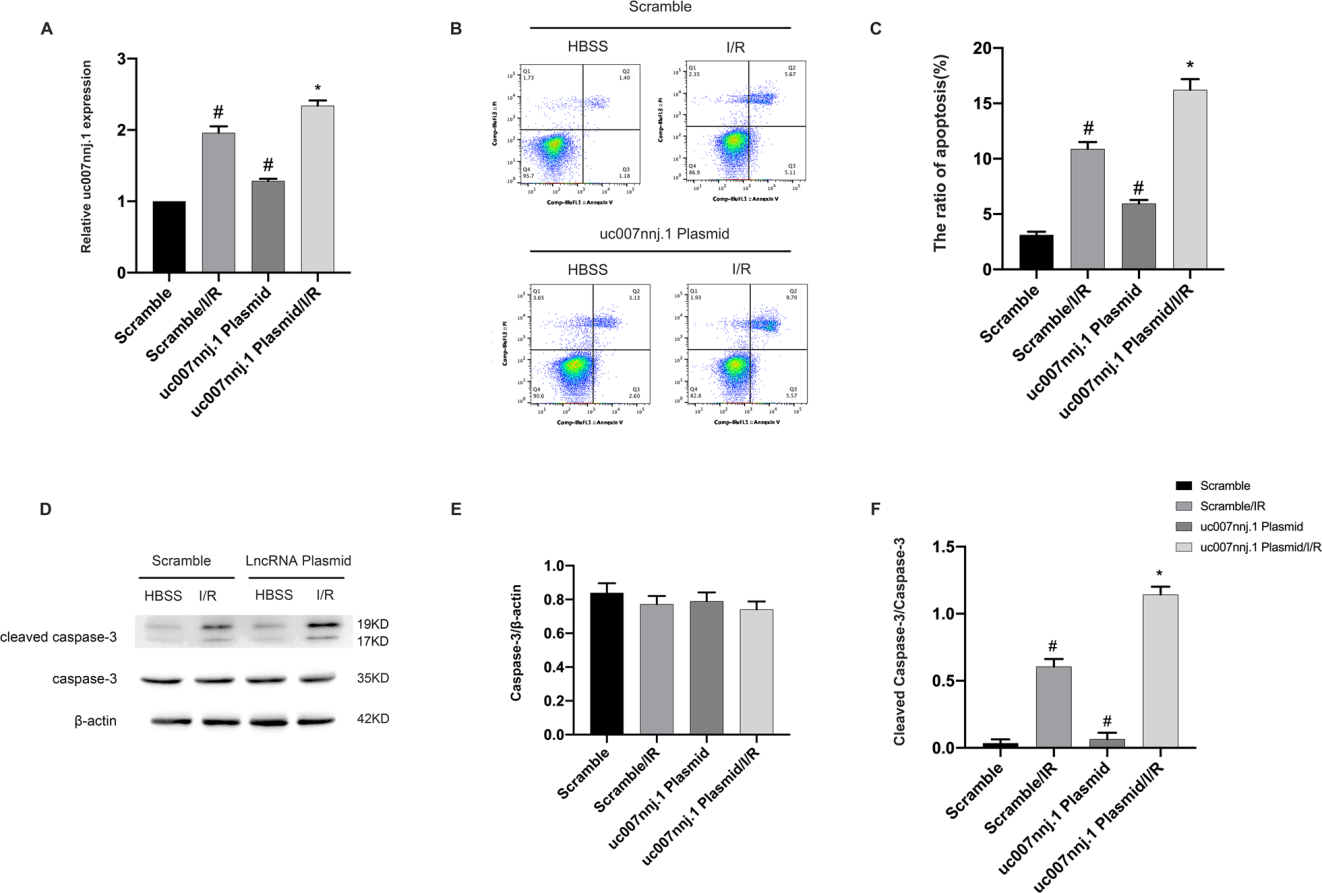
Overexpression of lncRNA uc007nnj.1 aggravated ischemia-induced neuronal apoptosis. RGCs were transfected with 1 g/mL lncRNA uc007nnj.1 plasmid or scramble and then treated with or without I/R. **A** RT-qPCR analysis of lncRNA uc007nnj.1 expression. **B**, **C** Representative data from the flow cytometric analysis of total cell apoptosis. The Q1 region represents necrotic cells, the Q2 region represents late apoptotic cells, the Q3 region represents early apoptotic cells, and the Q4 region represents normal cells. **D** Representative image showing a Western blot of caspase-3 and cleaved caspases-3. **E**, **F** Quantitative analysis of immunoreactive bands of caspase-3 (**E**) and cleaved caspase-3 (**F**). Data are expressed as mean ± SD (n = 6). ^#^*p* < 0.05, scramble with I/R group or lncRNA uc007nnj.1 plasmid group versus scramble group; *p < 0.05, lncRNA uc007nnj.1 plasmid with I/R group versus scramble with I/R group

The observation that lncRNA uc007nnj.1 deletion protected RGCs from apoptosis prompted us to investigate whether lncRNA uc007nnj.1 overexpression would aggravate the apoptosis induced by ischemia in vitro. We transfected RGCs with lncRNA uc007nnj.1 plasmid. RT-qPCR was used to analyze the efficiency of overexpression, and ischemia-induced lncRNA uc007nnj.1 expression was found to be significantly enhanced in RGCs (Fig. 3A). FCM results revealed that lncRNA uc007nnj.1 overexpression markedly aggravated ischemia-induced RGCs apoptosis (Fig. 3B, C). Furthermore, immunoblot analysis demonstrated that lncRNA uc007nnj.1 overexpression markedly augmented ischemia-induced accumulation of cleaved caspase-3 protein (Fig. 3D–F).

### lncRNA uc007nnj.1 mediates ischemia-induced RGCs apoptosis by regulating miR-155-5p as a ceRNA

**Fig. 4:**
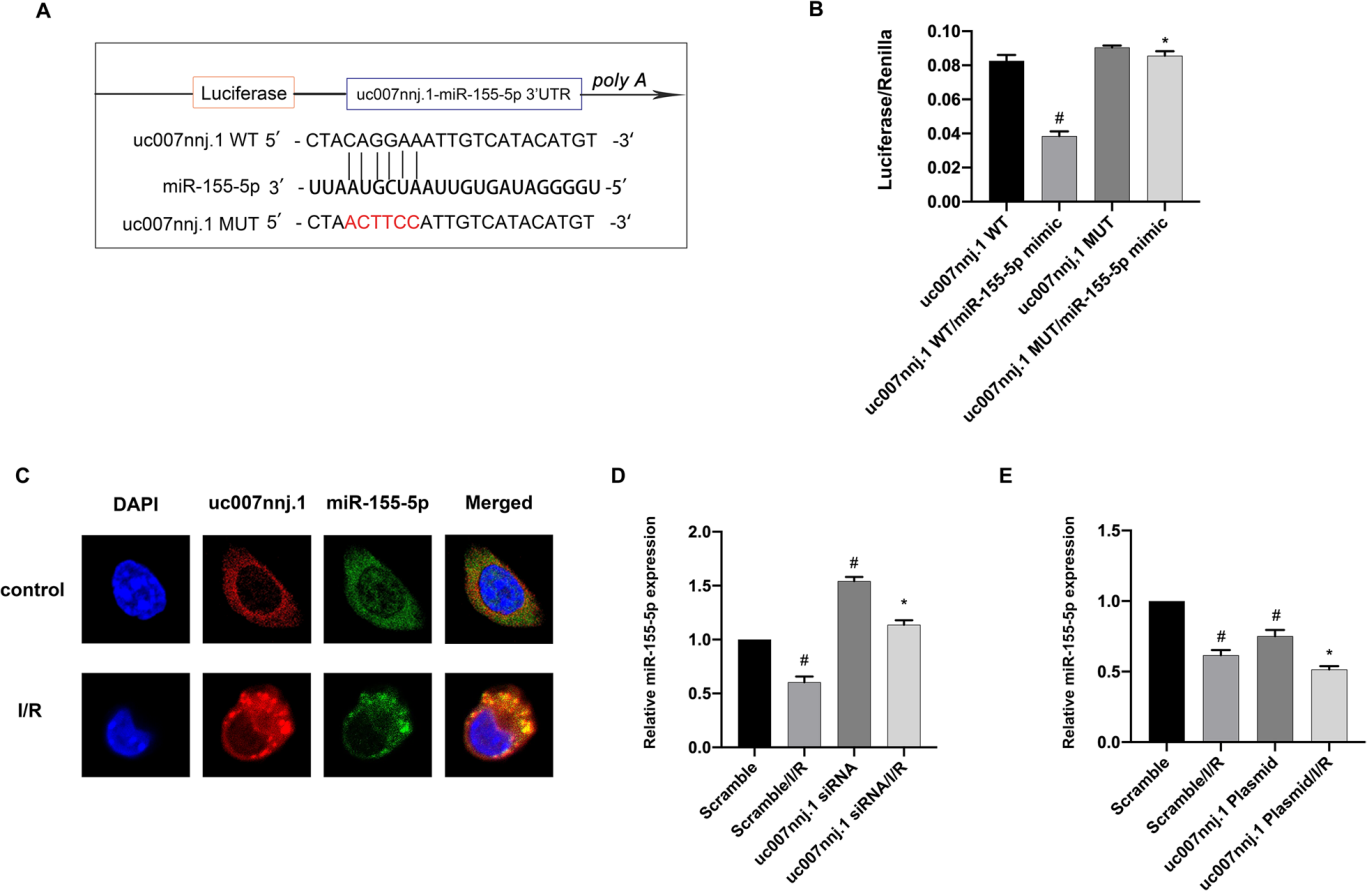
lncRNA uc007nnj.1 mediates ischemia-induced RGCs apoptosis by regulating miR-155-5p as a ceRNA. **A** An analysis of the sequence alignment revealed lncRNA uc007nnj.1 contained potential complementary binding sequences for miR-155-5p. **B** Luciferase activities were measured in RGCs after being co-transfected with lncRNA uc007nnj.1 WT or lncRNA uc007nnj.1 MUT with miR-155-5p mimic or scramble. **C** Intracellular colocalization of lncRNA uc007nnj.1 and miR-155-5p in RGCs treated with or without I/R damage. Scale bar: 10 µm. RGCs were transfected with 50 nmol/L lncRNA uc007nnj.1 siRNA or 1 g/mL lncRNA uc007nnj.1 plasmid or scramble and then treated with or without I/R damage. **D**, **E** RT-qPCR analyzed the expression of miR-155-5p. Data are expressed as mean ± SD (n = 6). ^#^*p* < 0.05, scramble with I/R group or lncRNA uc007nnj.1 siRNA group or lncRNA uc007nnj.1 plasmid group versus scramble group, lncRNA uc007nnj.1 WT/miR-155-5p mimic group versus lncRNA uc007nnj.1 WT group; *p < 0.05, lncRNA uc007nnj.1 siRNA with I/R group or lncRNA uc007nnj.1 plasmid with I/R group versus scramble with I/R group, lncRNA uc007nnj.1 MUT/miR-155-5p mimic group versus lncRNA uc007nnj.1 WT/miR-155-5p mimic group

Since the FISH results revealed that lncRNA uc007nnj.1 was predominantly localized in the cytoplasm (Fig. 1C), we wondered whether lncRNA uc007nnj.1 functions as a competitive endogenous RNA to mediate RGCs apoptosis. To test this idea, we applied in silico tools for the screening using the RegRNA database (http://regrna.mbc.nctu.edu.tw/index1.php) and eventually selected miR-155-5p as a potential miRNA decoy for lncRNA uc007nnj.1. Interestingly, sequence alignment analysis revealed that lncRNA uc007nnj.1 had potential binding sites for miR-155-5p (Fig. 4A). Luciferase activity results indicated that a miR-155-5p mimic inhibited luciferase activity when RGCs were cotransfected with the lncRNA uc007nnj.1 (WT) plasmid compared with the negative control or with the lncRNA uc007nnj.1 mutant plasmid (with mutation of the miR-155-5p binding site) (Fig. 4B). In addition, a FISH assay revealed that both lncRNA uc007nnj.1 and miR-155-5p are localized in the cytoplasm of RGCs (Fig. 4C). Furthermore, as detected by RT-qPCR, the downregulation of miR-155-5p could be reversed significantly by lncRNA uc007nnj.1 siRNA. In contrast, lncRNA uc007nnj.1 overexpression exerted the opposite effect (Fig. 4D, E). In conclusion, lncRNA uc007nnj.1 acts as a sponge for miR-155-5p.

### Ischemia-induced apoptosis was attenuated by miR-155-5p mimic

**Fig. 5:**
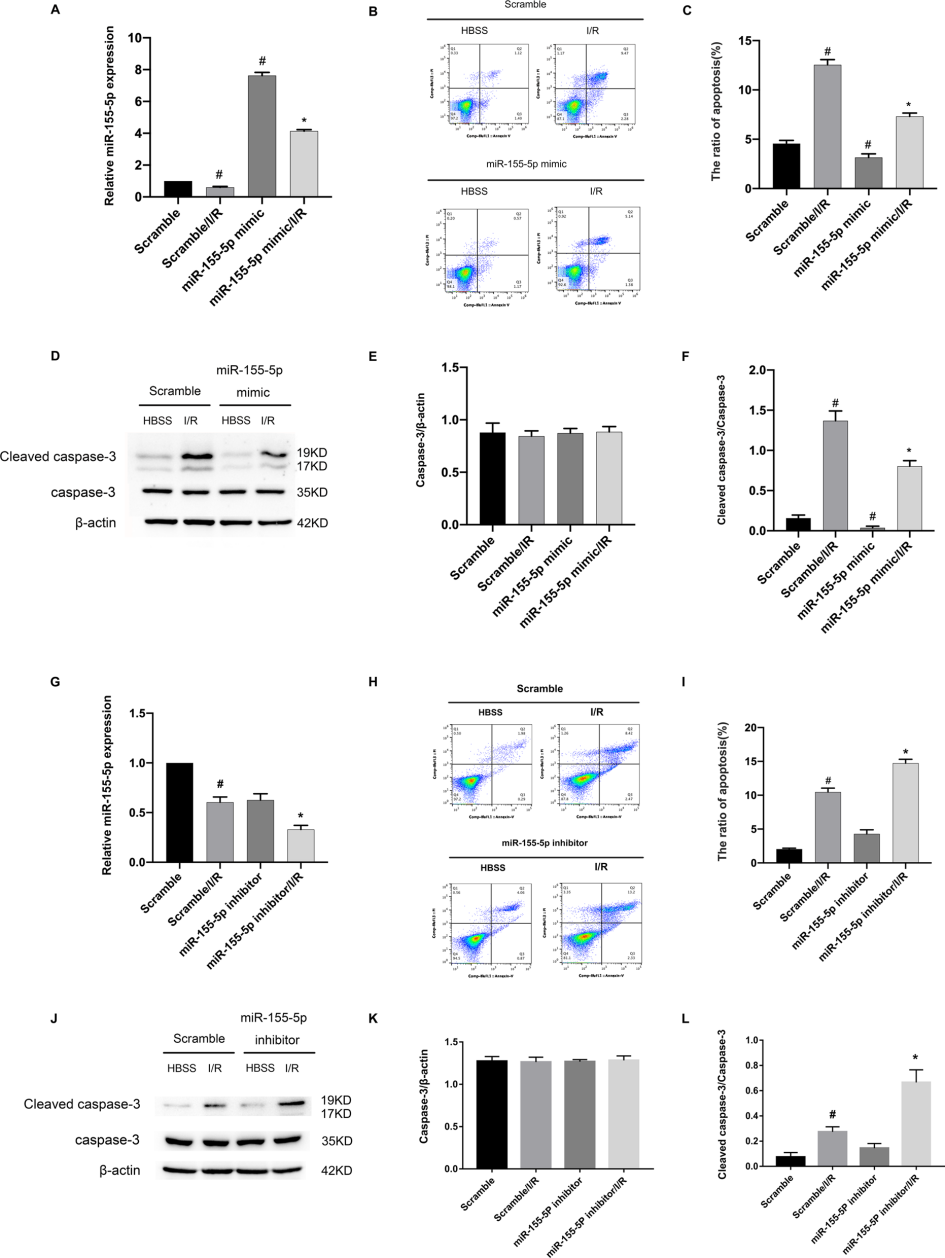
Ischemia-induced apoptosis was attenuated by miR-155-5p mimic. RGCs were transfected with 50 nM miR-155-5p mimic or miR-155-5p inhibitor or scramble and then with or without I/R treatment. **A** RT-qPCR analysis of miR-155-5p expression. **B**, **C** Representative data from the flow cytometric analysis of total cell apoptosis. The Q1 region represents necrotic cells, the Q2 region represents late apoptotic cells, the Q3 region represents early apoptotic cells, and the Q4 region represents normal cells. **D** Representative image showing a Western blot of caspase-3 and cleaved caspases-3. **E**, **F** Quantitative analysis of immunoreactive bands of caspase-3 (**E**) and cleaved caspase-3 (**F**). **G** RT-qPCR analysis of miR-155-5p expression. **H**, **I** Representative flow cytometric data and statistical data analysis of total cell apoptosis. **J**–**L** The apoptosis-related protein caspase-3 and cleaved caspase-3 were analyzed by Western blot. Data are expressed as mean ± SD (n = 6). ^#^*p* < 0.05, scramble with I/R group versus scramble group; *p < 0.05, miR-155-5p mimic with I/R group or miR-155-5p inhibitor with I/R group versus scramble with I/R group

Our data showed that miR-155-5p was a direct target of lncRNA uc007nnj.1, which prompted us to investigate the potential role of miR-155-5p in the pathogenesis of RGCs apoptosis under ischemic injury. First, RT-qPCR results demonstrated that the miR-155-5p mimic increased the levels of miR-155-5p in RGCs under basic and ischemic treatment (Fig. 5A). Second, flow cytometry revealed that the miR-155-5p mimic markedly attenuated I/R-induced total RGCs apoptosis (Fig. 5B, C). Immunoblotting results verified that the miR-155-5p mimic significantly inhibited the activity of caspase-3 (Fig. 5D–F). However, the effect was weakened by the miR-155-5p inhibitor (Fig. 5G–L). Collectively, these results indicate that miR-155-5p protects against RGCs apoptosis during ischemic treatment.

### Tle4 is a putative target of miR-155-5p

**Fig. 6:**
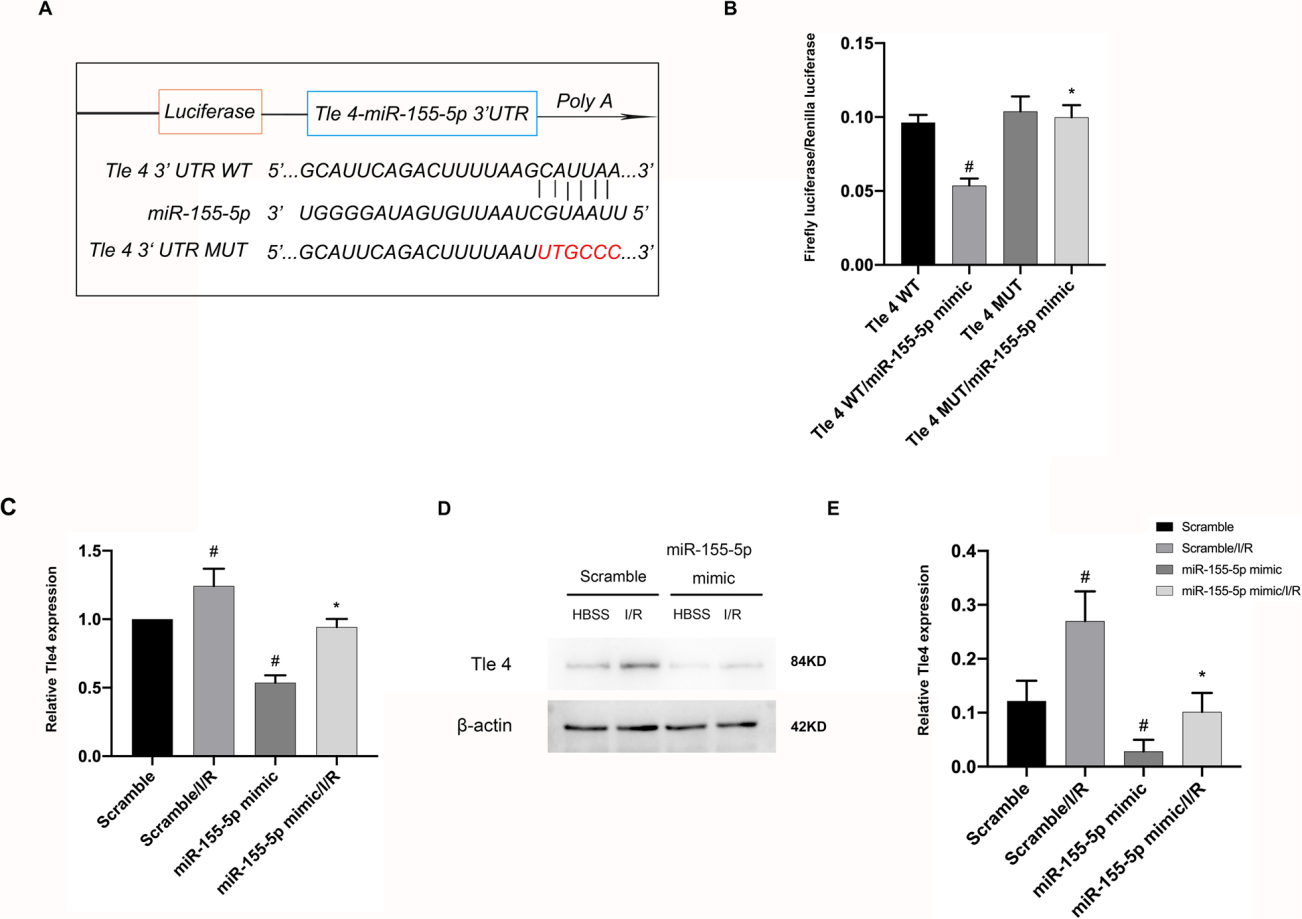
Tle4 is a putative target of miR-155-5p. RGCs were transfected with 50 nM miR-155-5p mimic, Tle4 siRNA, or scramble before I/R of 2/2 h. **A** The putative target site for miR-155-5p within the 3′-UTR of mouse Tle4. **B** Detection of luciferase activity after co-transfection with 3′-UTR luciferase reporter vector for mouse Tle4-WT, Tle4-MUT, and miR-155-5p mimic. **C**, **D** RT-qPCR and Western blot analysis of Tle4 and β-actin. **E** Densitometric analysis of immunoblot bands. Data are expressed as mean ± SD (n = 6). ^#^*p* < 0.05, scramble with I/R group or miR-155-5p mimic group versus scramble group, Tle4 WT/miR-155-5p mimic group versus Tle4 WT group; *p < 0.05*,* the miR-155-5p mimic group with I/R group versus scramble with I/R group, Tle4 MUT/miR-155-5p mimic group versus Tle4 WT/miR-155-5p mimic group

To identify the target gene regulated by miR-155-5p, TargetScan 7.2 was used (www.targetscan.org/mmu_72/), which indicated TLE Family Member 4 (Tle4) as a potential target for miR-155-5p with putative binding sites in the 3ʹ-UTR (Fig. 6A). Fragments of the Tle4 3′UTR were cloned into the luciferase gene, and RGCs were cotransfected with the construct and miR-155-5p mimic or miR-155-5p inhibitor. The results showed that the miR-155-5p mimic significantly reduced the luciferase activity in the Tle4 WT group, whereas mutation of the miR-155-5p binding site completely abolished the repression (Fig. 6B). Inversely, when the miR-155-5p inhibitor was bound to Tle4-WT rather than Tle4-MUT, the luciferase activity was significantly increased (Additional file 1: Fig. S1A). Meanwhile, the expression of Tle4 mRNA and protein was downregulated after transfection with miR-155-5p mimic while miR-155-5p inhibitor obviously promoted Tle4 mRNA and protein expression (Fig. 6C–E, Additional file 1: Fig. S1B–D).

### Inhibition of Tle4 ameliorated I/R-induced apoptosis of RGCs

**Fig. 7:**
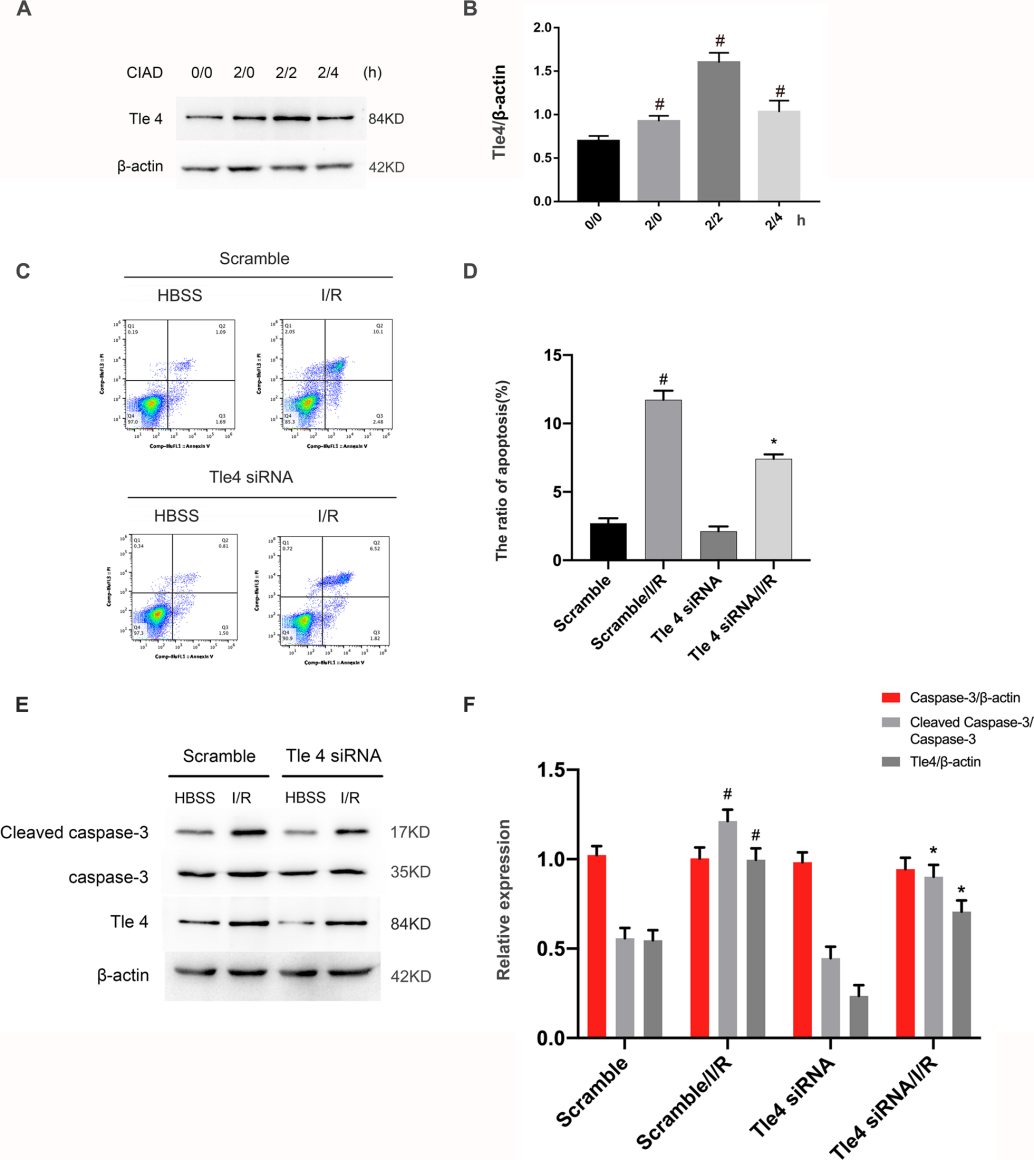
Inhibition of Tle4 ameliorated I/R-induced apoptosis of RGCs. RGCs were transfected with 50 nmol/L Tle4 siRNA or scramble and then treated with or without I/R damage. **A**, **B** RGCs were treated with or without I/R at indicated time points (ischemia/reperfusion for 0/0, 2/0, 2/2, 2/4 h, respectively). The expression levels of Tle4 were detected by Western blot. **C**, **D** Representative data from the flow cytometric analysis of total cell apoptosis. The Q1 region represents necrotic cells, the Q2 region represents late apoptotic cells, the Q3 region represents early apoptotic cells, and the Q4 region represents normal cells. **E**, **F** Representative image and quantitative analysis of Western blot of cleaved caspase-3, caspase-3, and Tle4. Data are expressed as mean ± SD (n = 6). ^#^*p* < 0.05, I/R for 0/0, 2/0, 2/2, 2/4 h versus 0/0 h, scramble with I/R group versus scramble group;^*^p < 0.05, Tle4 siRNA group with I/R group versus scramble with I/R group

Given that Tle4 is a potential target gene for miR-155-5p, we further investigated the role of Tle4 in ischemia-induced RGCs apoptosis. After transfection with Tle4 siRNA or scramble, RGCs were treated with or without I/R damage (ischemia for 2 h, reperfusion for 2 h). Immunoblotting showed that the expression of Tle4 was increased at 0 h after reperfusion, peaked at 2 h, and then was reduced at 4 h after reperfusion (Fig. 7A, B). FCM analysis revealed that knockdown of Tle4 significantly reduced apoptosis in RGCs (Fig. 7C, D). Immunoblot analysis verified that Tle4 knockdown by siRNA markedly suppressed I/R-induced expression of Tle4 and the activity of caspase-3 (Fig. 7E, F). Collectively, the data suggest that Tle4 is an inducer of apoptosis.

### lncRNA uc007nnj.1 knockdown attenuated ischemia-induced RGCs apoptosis via miR-155-5p

**Fig. 8:**
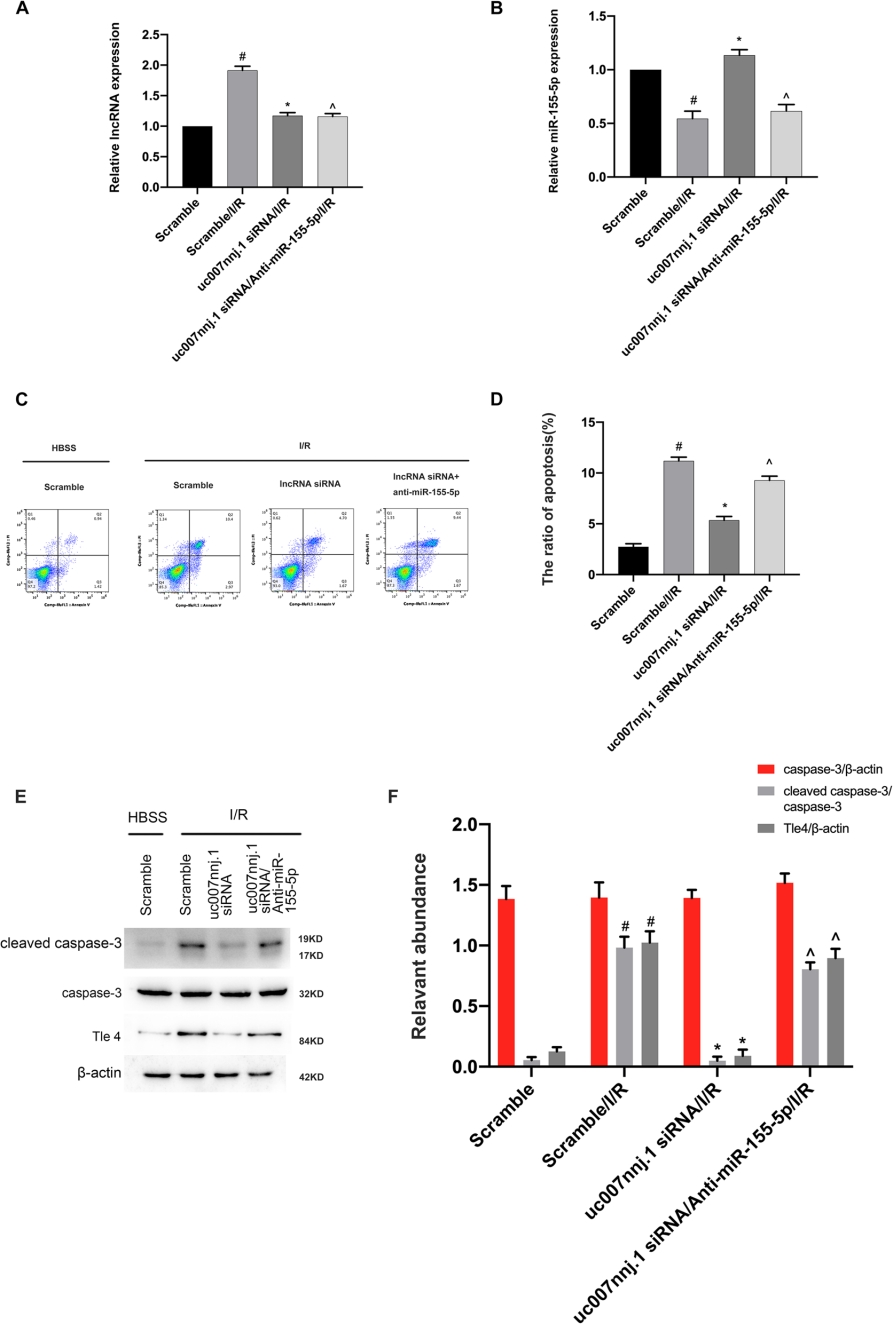
lncRNA uc007nnj.1 knockdown attenuated ischemia-induced RGCs apoptosis via miR-155-5p. RGCs were co-transfected with 50 nM lncRNA uc007nnj.1 siRNA and anti-miRNA-155-5p or scramble and then treated with or without I/R. **A**, **B** RT-qPCR analysis of the expression levels of lncRNA uc007nnj.1 and miR-155-5p. **C**, **D** FCM analysis of total RGCs apoptosis. The Q1 region represents necrotic cells, the Q2 region represents late apoptotic cells, the Q3 region represents early apoptotic cells, and the Q4 region represents normal cells. **E**, **F** Western blot analysis for detection of activated caspase-3, caspase-3, and Tle4. Data are expressed as mean ± SD (n = 6). ^#^*p* < 0.05, scramble with I/R group versus scramble group; *p < 0.05, lncRNA uc007nnj.1 siRNA with I/R group versus scramble with I/R group; ^^^*p* < 0.05, lncRNA uc007nnj.1 siRNA/Anti-miR-155-5p with I/R group versus lncRNA uc007nnj.1 siRNA with I/R group

We further investigated whether miR-155-5p mediated the proapoptotic effect of lncRNA uc007nnj.1. RGCs were transfected with lncRNA uc007nnj.1 siRNA and miR-155-5p inhibitor. The transfection efficiency was confirmed by RT-qPCR analysis (Fig. 8A, B). Flow cytometry and Western blot analyses were performed using the same transfected cells. The results indicated that lncRNA uc007nnj.1 deletion attenuated RGCs apoptosis and suppressed ischemia-induced expression of Tle4 and the activity of caspase-3. Intriguingly, this effect was reversed by miR-155-5p knockdown (Fig. 8C–F).

### Suppression of lncRNA uc007nnj.1 ameliorated I/R-induced mouse retinal damage via miR-155-5p/Tle4 axis regulation

**Fig. 9:**
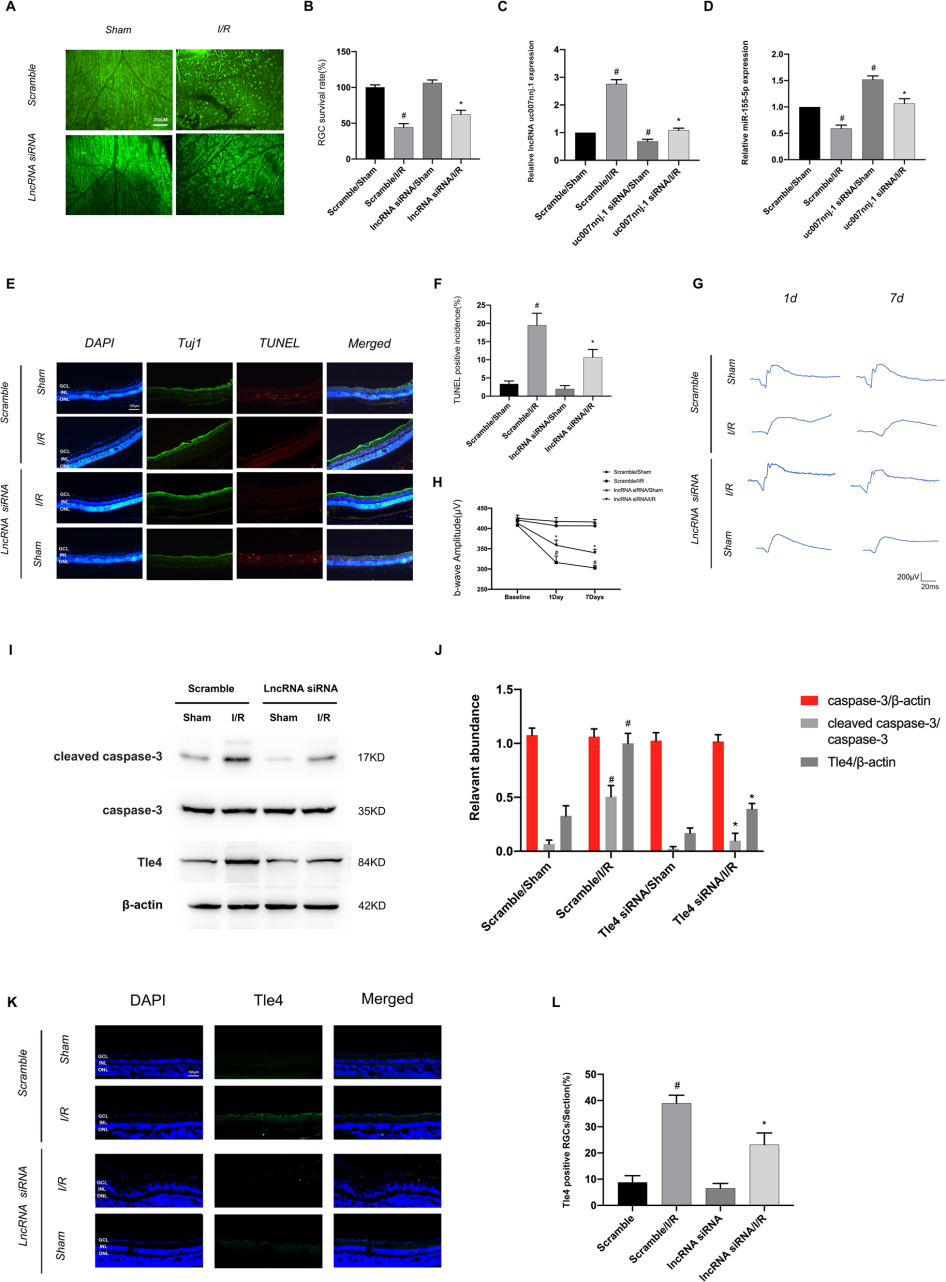
Suppression of lncRNA uc007nnj.1 ameliorated I/R-induced mouse retinal damage via miR-155-5p/Tle4 axis regulation. lncRNA uc007nnj.1 siRNA or scramble (1 μg/μL) was given to C57BL/6J mice vitreous 24 h before retinal I/R injury. **A**, **B** Representative immunolabeled images and quantitative analysis for anti-Tuj1 as RGCs marker in a magnified area of flat-mount retina 7 days after I/R treatment. Scale bar: 100 μm. **C**, **D** RT-qPCR analysis shows lncRNA uc007nnj.1 and miR-155-5p levels in retina harvested from I/R and sham group mice injected with scrambled siRNA or lncRNA uc007nnj.1 siRNA. **E** Representative images of double staining with Tuj1 antibody (green) and TUNEL (red) reagents on retinal sections. **F** Comparison of the ratio for the density of TUNEL positive RGCs to the total number of DAPI-stained nuclei in the ganglion cell layer in a different group. Scale bar: 100 μm. **G** Representative scotopic ERG traces under the intensity of 3.0 cd·s/m^2^ on day 1 and day 7 after I/R injury. **H** Statistical analysis of the b-wave amplitudes at 3.0 cd·s/m^2^ under dark-adapted conditions. **I**–**J** Representative Western blot images and densitometric measurements show the levels of cleaved caspase-3, caspase-3 and Tle4. **K**, **L** Representative immunolabeled images and quantitative analysis of Tle4 (green) on the RGC layer. Scale bar: 100 μm. Data are expressed as mean ± SD (n = 6). ^#^*p* < 0.05, scramble/I/R group versus scramble/sham group; *p < 0.05, lncRNA uc007nnj.1 siRNA/I/R group versus scramble/I/R group

Thus, as a whole, our data indicate that lncRNA uc007nnj.1 silencing protects against ischemia-induced RGCs apoptosis; we aimed to further elucidate the molecular mechanism in vivo by evaluating the effect of lncRNA uc007nnj.1 in I/R-induced mouse retinal damage. The data showed that intravitreous preinjection of lncRNA uc007nnj.1 siRNA 24 h before retinal I/R injury prevented a reduction in the intensity of Tuj 1-positive cell staining (Fig. 9A, B). RT-qPCR analysis showed that lncRNA uc007nnj.1 siRNA significantly reduced the lncRNA uc007nnj.1 level and increased miR-155-5p expression under basic and I/R treatment conditions (Fig. 9C, D). TUNEL staining revealed that lncRNA uc007nnj.1 siRNA markedly reduced the I/R-induced apoptosis in RGCs (Fig. 9E, F). Moreover, scotopic ERG demonstrated that knockdown of lncRNA uc007nnj.1 significantly prevented I/R-induced b-wave decline (Fig. 9G, H). In addition, Western blot analysis demonstrated that knockdown of lncRNA uc007nnj.1 decreased the I/R-induced increase in cleaved caspase-3 and Tle4 (Fig. 9I, J). Immunofluorescence staining further showed that preoperative injection of lncRNA uc007nnj.1 significantly decreased the expression of Tle4 in I/R induced retina, particularly in the ganglion cell layer (Fig. 9K, L). Additionally, we further analyze the RGCs apoptosis in mice models with high lncRNA uc007nnj.1 expression as compared to the control group. Importantly, Immunofluorescence staining, ERG, and Western blot data indicated overexpression of lncRNA uc007nnj.1 promoted I/R induced RGCs apoptosis and visual impairment (Additional file 1: Fig. S2). Collectively, silencing of lncRNA uc007nnj.1 might confer protective effects against I/R-induced damage in the retina through miR-155-5p/Tle4 axis regulation.

## Discussion

**Fig. 10:**
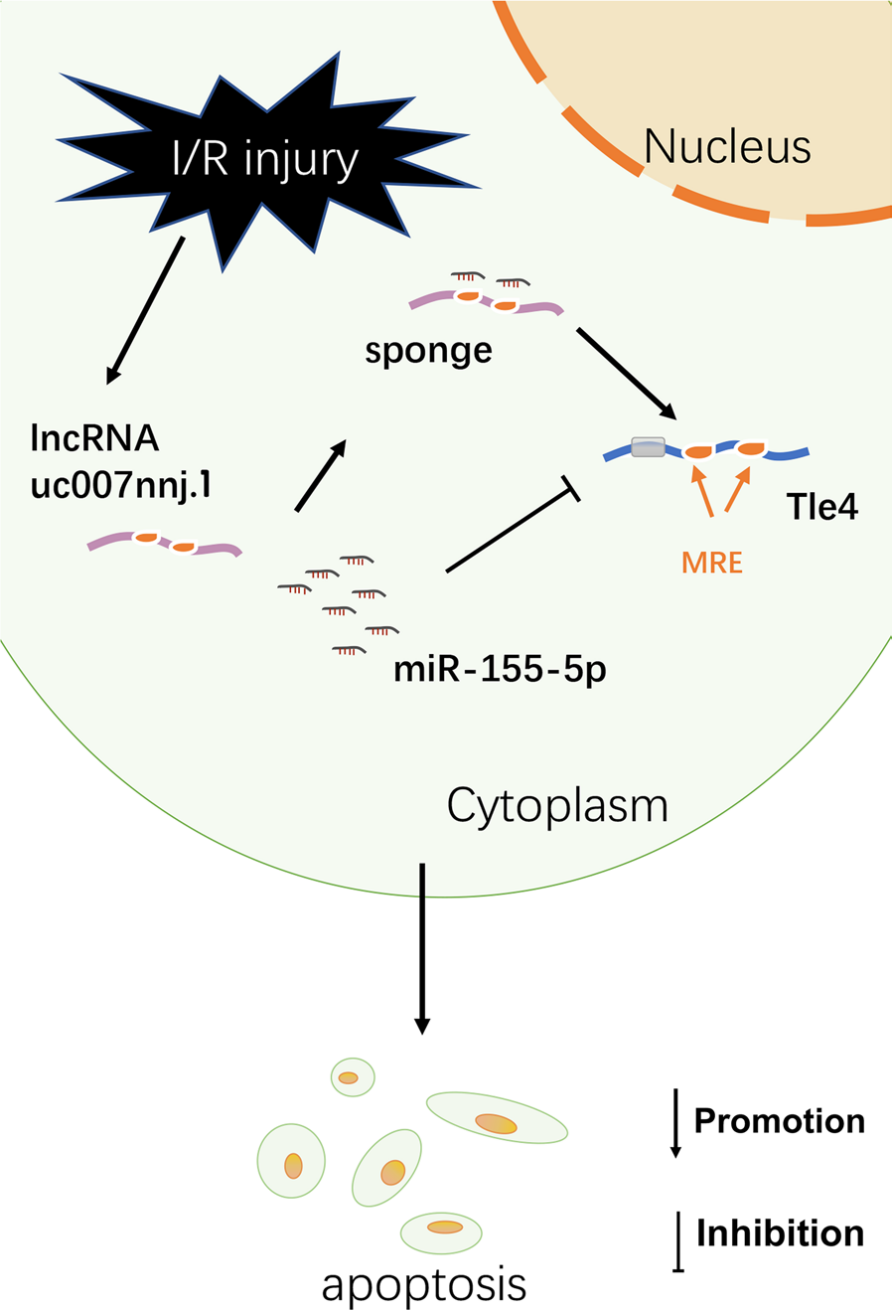
The interaction mechanism diagram

Apoptosis of retinal ganglion cells (RGCs) is a pivotal aspect of irreversible blindness caused by ischemic injury. lncRNAs have been reported to be involved in a wide variety of physiological and pathological processes in apoptosis (Zhao et al. 2020; Xiong et al. 2021). Based on our lncRNA chip data, several highly expressed lncRNAs were selected for RT-qPCR verification (Ge et al. 2020). Meanwhile, we investigated the functional stability of candidate lncRNAs in an in vitro model of ischemic injury. In a previous study, we revealed the proapoptotic function of one of the candidate lncRNAs, Ttc3-209, which was found to act via the ceRNA pathway in a mouse model of I/R damage. To further analyze the role and mechanism of apoptosis-related lncRNAs in RGCs, we selected lncRNA uc007nnj.1 for further study (fold change greater than 8). This study revealed both in vivo and in vitro that lncRNA uc007nnj.1 is crucial in apoptosis induction in I/R-injured RGCs. Mechanistically, lncRNA uc007nnj.1 mediated mouse neuronal apoptosis induced by I/R by sponging miR-155-5p and thus targeting the regulation of Tle4. We present a specific schematic diagram of our research in Fig. 10.

Several studies have reported that lncRNAs are critical players in a variety of ocular problems (Zhang et al. 2019). For instance, classically, lncRNA-MALAT1 and lncRNA-MEG3 can inhibit RGCs apoptosis by promoting the PI3K/Akt signaling pathway. In contrast, lncRNA Ttc3-209 and TUG1 mediate neural cell apoptosis, including that of RGCs (Billingham 1994; Millar and Penrose 1980). In the current study, we investigated lncRNA uc007nnj.1, which was upregulated in retinal ischemia–reperfusion and located in the cytoplasm of RGCs. RGCs degeneration is the most common event in retinal dysfunction. lncRNA uc007nnj.1 silencing decreases I/R-induced RGCs apoptosis. On the other hand, enhancing the expression of lncRNA uc007nnj.1 aggravates this event. Thus, empirical analysis suggests that lncRNA uc007nnj.1 plays a critical role in I/R injury.

More interestingly, the cross-regulatory network between miRNAs and lncRNAs has recently been identified. Many studies have reported that lncRNAs can act as sponges of miRNAs to regulate their targeted genes (Ge et al. 2019; Yan et al. 2015; Wang et al. 2017; Zhang et al. 2018). Health letter analysis revealed that miR-155-5p can directly bind to lncRNA uc007nnj.1. To strengthen this assumption, several experiments were performed. First, dual-luciferase reporter assays confirmed that lncRNA uc007nnj.1 interacted with miR-155-5p. Second, RNA-FISH colocalization assays indicated that lncRNA uc007nnj.1 interacts with miR-155-5p in the cytoplasm of RGCs. Finally, RT-qPCR data showed that I/R injury suppresses the expression of miR-155-5p, which was enhanced by overexpression of lncRNA uc007nnj.1; in contrast, lncRNA uc007nnj.1 silencing reversed this effect. Finally, we found that miR-155-5p mediates the pro-apoptotic function of lncRNA uc007nnj.1. Notably, the present study established that lncRNA uc007nnj.1 is a damaging factor in ischemic injury, whereas miR-155-5p is a protective miRNA in ischemic injury. lncRNA uc007nnj.1 sponged the miR-155-5p to induce apoptosis in RGCs during ischemic injury.

The role of miR-155-5p remains controversial. One study reported that miR-155-5p contributes to the suppression of osteosarcoma cell death (Bhattacharya et al. 2016). Conversely, another study showed that miR-155-5p inhibited apoptosis in HCC by directly targeting the 3′-UTR of PTEN (Fu et al. 2017). For the first time, our results showed that miR-155-5p mimic notably suppressed the I/R-induced RGCs apoptosis in vitro. Interestingly, we verified that Tle4 is a direct target of miR-155-5p through in silico prediction, luciferase reporter assay, RT-qPCR, and immunoblotting analyses. However, the role of Tle4 in apoptosis seems very small. Only one has study revealed that forced Tle4 expression caused apoptosis and cell death in myeloid leukemia, which is consistent with our current findings that Tle4 knockdown attenuated I/R-induced apoptosis of RGCs. Finally, we verified that the lncRNA uc007nnj.1/miR-155-5p/Tle4 axis mediated I/R-induced RGCs apoptosis and the resulting decline in visual function in C57BL/6J mice.

## Conclusion

Our research provides evidence of a specific signaling pathway involved in retinal neuronal cell damage induced by I/R injury in mice. We revealed that lncRNA uc007nnj.1 promotes RGCs apoptosis in vitro* and *in vivo during ischemic injury. Mechanistically, lncRNA uc007nnj.1 sponges miR-155-5p to upregulate the expression of Tle4. Thus, identifying the ceRNA regulatory network would provide novel insight into the mechanisms of retinal ischemia–reperfusion and ultimately facilitate the development of lncRNA-directed diagnostics and therapeutics.

## Supplementary Information

**Additional file 1: Table S1.** Upregulated lncRNAs in Scramble/I/R group vs. Scramble group. **Table S2.** Primers of genes. **Figure S1.** Tle4 is a putative target of miR-155-5p. **Figure S2.** lncRNA uc007nnj.1 expression accelerated the process of I/R-induced RGCs apoptosis in vivo.

## Data Availability

All data generated or analyzed during this study are included in this published article.

## Abbreviations

I/R: Ischemia–reperfusion
RGCs: Retinal ganglion cells
lncRNAs: Long noncoding RNAs
ceRNAs: Competing endogenous RNA
Tle4: TLE family member 4
siRNA: Small interfering RNA
FISH: Fluorescence in situ hybridization
FCM: Flow cytometry
RT-qPCR: Reverse transcription quantitative real-time PCR
ERG: Electroretinography
