# Supplementary material for: Long noncoding RNA uc007nnj.1 mediates neuronal death induced by retinal ischemia/reperfusion in mice via the miR-155-5p/Tle4 axis
Source: Mol Med. 2023 Jan 18;29:9. doi: 10.1186/s10020-022-00591-5 (PMC9850566; doi:10.1186/s10020-022-00591-5)
Supplement: Supplementary file 1 — Additional file 1: Table S1. Upregulated lncRNAs in Scramble/I/R group vs. Scramble group. Table S2. Primers of genes. Figure S1. Tle4 is a putative target of miR-155-5p. Figure S2. lncRNA uc007nnj.1 expression accelerated the process of I/R-induced RGCs apoptosis in vivo. [file 10020_2022_591_MOESM1_ESM.docx]

**Additional Materials**

**Long noncoding RNA uc007nnj.1** **mediates neuronal death induced by retinal ischemia/reperfusion in mice via the miR-155-5p/Tle4 axis**

Yuqing Feng, Jinfang Lu, Xujun Peng, Yanni Ge, Ran Zhang^,^ and Huiling Li

**Additional Table S1.**

| **Seqname** | **I/R group vs. Scramble group upregulation Fold change** | **Nucleotides(nt)** |
| --- | --- | --- |
| ENSMUST00000135399 | 8.8036311 | 2723 |
| AK082454 | 8.8005763 | 3932 |
| AK013121 | 8.7506446 | 1169 |
| **uc007nnj.1** | **8.586762** | **1669** |
| ENSMUST00000176215 | 8.5860049 | 477 |
| AK048998 | 8.5241797 | 2188 |
| uc.19- | 8.4993599 | 256 |
| ENSMUST00000153863 | 8.4294728 | 599 |
| uc.74- | 8.4155082 | 537 |
| AK085151 | 8.3974133 | 3745 |

**Additional Table S2. primers of genes**

| **Gene name** | **Forward primer (5’ to 3’)** | **Reverse primer (5’ to 3’)** | **RT primer (5’ to 3’)** |
| --- | --- | --- | --- |
| lncRNA uc007nnj.1 | GCTCTTGACTGTGCGTCTCC | GCTGCGACAAGCTAGCAGAT | / |
| miR-155-5p | GCGCGTTAATGCTAATTGTGAT | AGTGCAGGGTCCGAGGTATT | GTCGTATCCAGTGCAGGGTCCGAGGTATTCGCACTGGATACGACACCCCT |
| Tle4 | CCATCAGCCAGTTTCCGAGGTG | GCGTGTCTTGTCTAGGCCAT | / |
| β-actin | GCTATGCTCTCCCTCACG | ACGCACGATTTCCCTCT | / |
| U6 | AGAGAAGATTAGCATGGCCCCTG | AGTGCAGGGTCCGAGGTATT | GTCGTATCCAGTGCAGGGTCCGAGGTATTCGCACTGGATACGACAAAAAT |

**The identification of retinal ganglion cells(RGCs)**


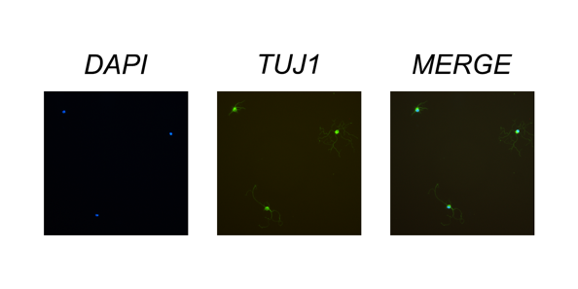


Immunofluorescence was performed using an antibody against Tuj1(green) to identify primary retinal ganglion cells(RGCs). Nuclei were counterstained with DAPI(blue). Scale bar: 50µm.


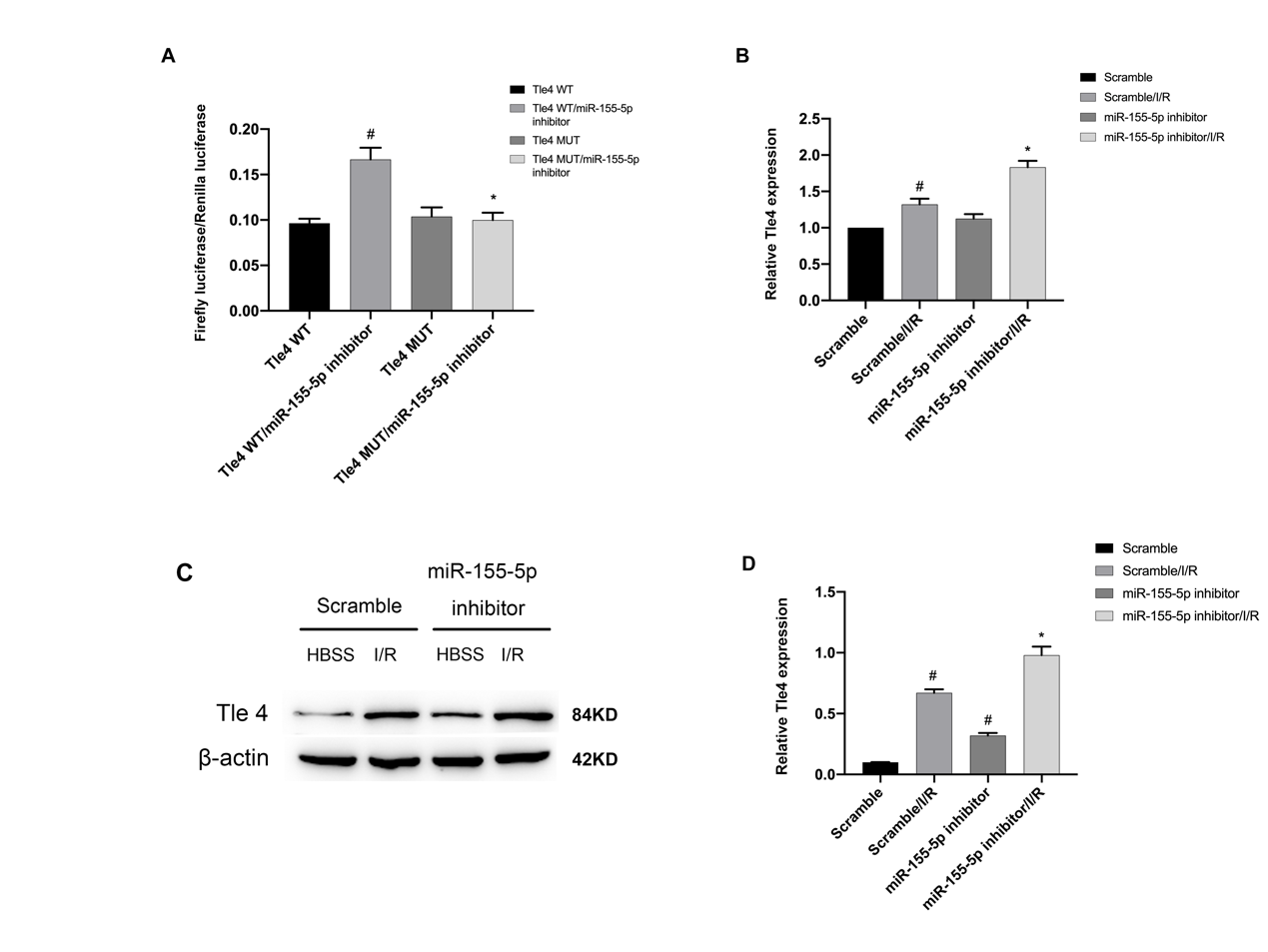


**Additional Figure S1.** **Tle4 is a putative target of miR-155-5p**

RGCs were transfected with 50nM miR-155-5p inhibitor or scramble before I/R of 2/2h. (A) Detection of luciferase activity after co-transfection with 3’-UTR luciferase reporter vector for mouse Tle4-WT, Tle4-MUT, and miR-155-5p inhibitor. (B-C) RT-qPCR and western blot analysis of Tle4 and β-actin. (D) Densitometric analysis of immunoblot bands. Data are expressed as mean ± SD(n=6). ^#^*p<0.05*, scramble with I/R group or miR-155-5p inhibitor group versus scramble group, Tle4 WT/miR-155-5p inhibitor group versus Tle4 WT group; ^*^*p<0.05,* the miR-155-5p inhibitor group with I/R group versus scramble with I/R group, Tle4 MUT/miR-155-5p inhibitor group versus Tle4 WT/miR-155-5p inhibitor group.


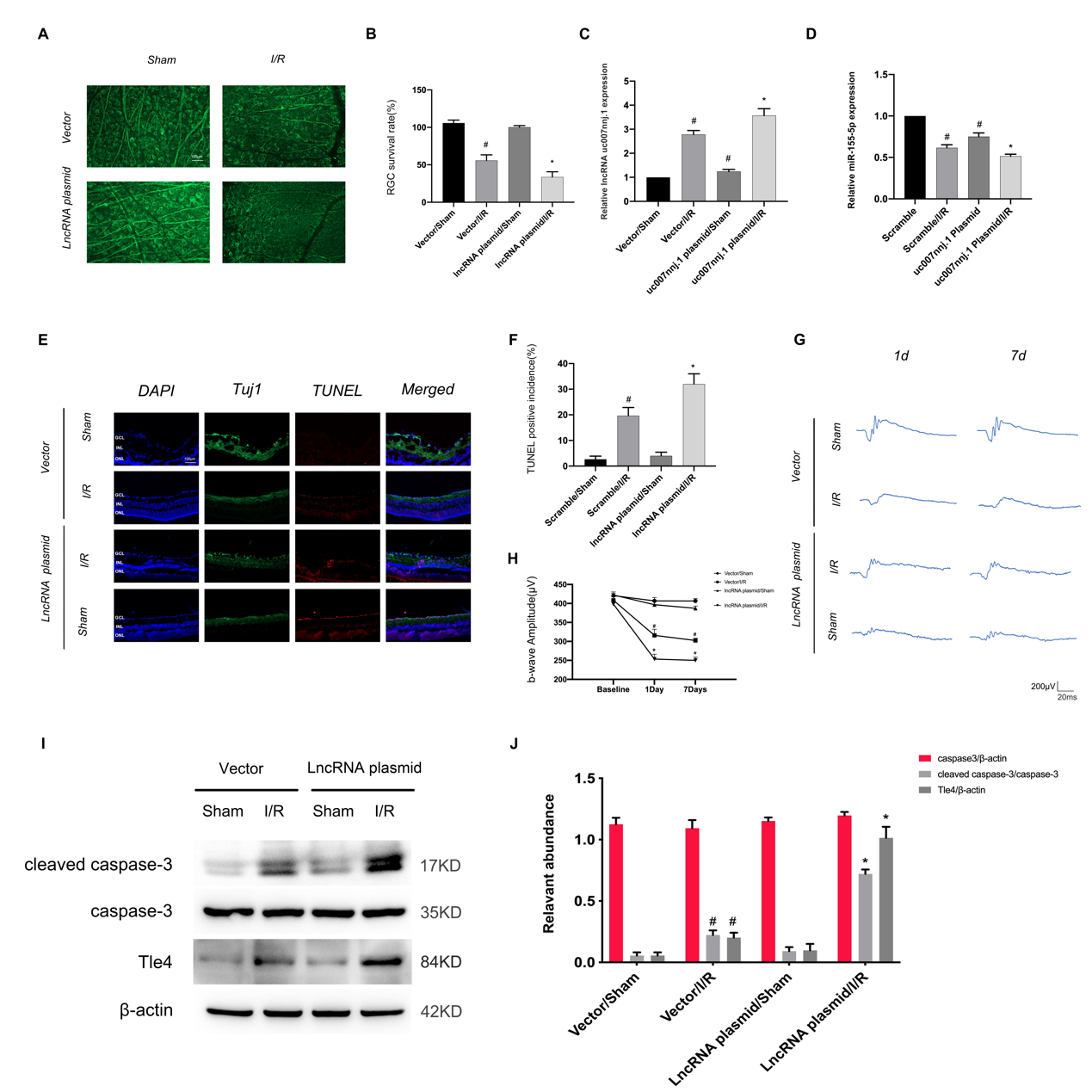


**Additional Figure S2. lncRNA uc007nnj.1 expression accelerated the process of I/R-induced RGCs apoptosis *in vivo***

lncRNA uc007nnj.1 plasmid or Vector (1 μg/μL) was given to C57BL/6J mice vitreous 24 hours before retinal I/R injury. (A-B) Representative immunolabeled images and quantitative analysis for anti-Tuj1 as RGCs marker in a magnified area of flat-mount retina 7 days after I/R treatment. Scale bar: 100 μm. (C-D) RT-qPCR analysis shows lncRNA uc007nnj.1 and miR-155-5p levels in retina harvested from I/R and sham group mice injected with Vector or lncRNA uc007nnj.1 plasmid. (E) Representative images of double staining with Tuj1 antibody(green) and TUNEL(red) reagents on retinal sections. (F) Comparison of the ratio for the density of TUNEL positive RGCs to the total number of DAPI-stained nuclei in the ganglion cell layer in a different group. Scale bar: 100 μm. (G) Representative scotopic ERG traces under the intensity of 3.0 cd·s/m^2^ on day 1 and day 7 after I/R injury. (H) Statistical analysis of the b-wave amplitudes at 3.0 cd·s/m^2^ under dark-adapted conditions. (I-J)Representative Western blot images and densitometric measurements show the levels of cleaved caspase-3、caspase-3 and Tle4. Data are expressed as mean ± SD (n = 6). ^#^*p<0.05*, Vector/I/R group versus Vector/sham group; ^*^*p<0.05*, lncRNA uc007nnj.1 plasmid/I/R group versus Vector/I/R group.
